# Parental perspectives on children’s screen use: Exploring impact, challenges, and support needs - A qualitative study

**DOI:** 10.1177/22799036261462200

**Published:** 2026-06-17

**Authors:** Marie Werner, Felicia Delfechresh, Aleksandra Barstowe, Sabina Kapetanovic, Emma Claesdotter-Knutsson

**Affiliations:** 1 Department of Clinical Sciences Lund, 59568 Faculty of Medicine Lund University, Lund, Sweden; 2 Department of Child and Adolescent Psychiatry, 59564 Skåne University Hospital, Lund, Sweden; 3 Department of Social and Behavioural Studies, 42749 University West, Trollhättan, Sweden

**Keywords:** family, children, screen use, gaming, social media, support, intervention

## Abstract

**Background:**

Like adults, many children and adolescents spend several hours each day engaged in screen-based activities. This has raised concerns among adults, and the consequences of screen use are the subject of intense research and public debate. Despite growing research interest, there is currently a lack of evidence-based interventions available to families experiencing negative consequences related to their child’s screen use. The aim of this study was to explore parents’ perceptions of the consequences of their children’s screen use, as well as the type of support they seek in response.

**Design and Methods:**

Through collaboration with schools in a medium-sized Swedish city, eleven participants with children aged 12–17 were recruited. Semi-structured interviews were conducted, transcribed, and analyzed using thematic analysis.

**Results:**

The analysis resulted in three main themes: *Consequences of children’s screen use*, *A problem for the whole society*, and *Need of support on family and individual level*, along with a total of nine subthemes.

**Conclusion:**

Our conclusion is that, according to parents, screen use among children is a complex phenomenon that calls for broad and systemic responses. While consequences for the child are primarily observed at the microsystem level—affecting relationships, health, and development—parents also express expectations for change at higher societal levels. Taken together, our findings underscore the need for multi-level interventions that combine structural measures with accessible, family-oriented support strategies aimed at promoting healthy digital habits in children and adolescents.

## Introduction

Digital technologies such as computers, smartphones and tablets have become an integral part of children’s and adolescents’ daily lives.^1^ Screen use refers to the general time spent on digital devices with screens (e.g., smartphones, tablets, computers, televisions) for any purpose. Gaming specifically denotes playing video or computer games, whether online or offline. Social media use involves engaging with platforms designed for social networking, content sharing, and communication (e.g., TikTok, Instagram, Snapchat).^2^ While these activities often overlap, they represent distinct behaviors with potentially different impacts and parental concerns.^3^ Recent Swedish data reveal that nearly all individuals aged 8 to 19 have accessed the internet, with 98% engaging with some form of social media in the past year, 97% using it weekly, and 91% engaging daily. Social media usage is even higher among middle and high school students, with 100% of adolescents reporting daily engagement.^4^ Notably, gender differences exist in screen usage patterns, with boys being five times more likely to participate in video gaming than girls, while girls demonstrate a greater preference for social media platforms.^5^ Among 13-year-old boys, nearly all report playing video games for more than three hours per day.^6^ The use of digital media in itself is not problematic, and reviews of the scientific literature show that factors such as amount of time, content, context, and individual characteristics all play a role in whether children experience negative consequences of screen use or not.^2^ According to Swedish national recommendations, children aged 13–18 years should not engage in screen use exceeding three hours per day.^7^ However, national surveys in which adolescents have been asked to estimate their own screen time indicate substantially higher levels, with a total daily use of approximately six to seven hours.^8^

With the increasing prevalence of digital technologies, growing concerns have emerged among parents and professionals working with children and adolescents in educational, municipal, and healthcare settings regarding the potential effects of screen use on young people’s well-being and development.^9^ Research has demonstrated that excessive screen use is associated with both psychological and physical health concerns^10^ and may contribute to the development of gaming disorder.^11^ Gaming disorder was officially included in the 11th revision of the International Classification of Diseases (ICD-11) by the World Health Organization (WHO) in June 2018, and is characterized by impaired control over gaming, prioritization of gaming over other activities, and continued engagement despite negative consequences.^12^ Similarly, internet gaming disorder is included in the Diagnostic and Statistical Manual of Mental Disorders, Fifth Edition (DSM-5), though it is not formally recognized as a clinical diagnosis but rather as a condition warranting further research.^13^ Despite the identified risks associated with extensive video gaming, there are also studies that highlight several benefits of video game use, including enhanced language acquisition,^14^ improved metacognitive abilities,^15^ and strengthened cognitive and problem-solving skills in children.^16^

The digital environment provides children and adolescents with unprecedented access to diverse content through various technological devices, including computers, smartphones, tablets, and gaming consoles.^17^ This widespread media integration has contributed to a complex and often challenging landscape, not only for parents and healthcare professionals but also for young individuals themselves. Empirical studies have identified a concerning association between frequent social media use and mental health issues such as eating disorders, depression, and body image disturbances.^18,19^ Additionally, the risk of cyberbullying remains a significant concern, with research linking it to increased instances of self-harm and suicidal behaviors.^20^ The vast accessibility of online content presents further risks, as exposure to harmful material can have detrimental effects on impressionable youth. Adolescents engaging in risky behaviors are particularly vulnerable, with social media exposure often correlating with substance and alcohol use.^21^ Moreover, excessive social media use has been associated with addictive tendencies that contribute to heightened stress, anxiety, and disrupted sleep patterns.^22,23^ In the long term, these patterns may lead to diminished social competence and an increased risk of severe mental health and socioemotional challenges.^1,24^

Currently, no formal diagnosis exists for excessive social media use or internet addiction, nor are there standardized treatment protocols for problematic computer gaming.^25^ In Sweden, there is an urgent need for a collaboration between child and adolescent psychiatry departments, student health services and social services to develop intervention programs addressing problematic screen use and gaming to mitigate future risks of psychopathology and developmental impairments.^9,26^

Bronfenbrenner’s bioecological model^27^ demonstrates that children’s development is influenced by both close relationships and broader societal structures. It highlights the importance of the interaction between the individual and their environment and emphasizes that development is a dynamic process that changes over time. Bronfenbrenner’s theoretical model of environmental influences on child development has been further developed by Johnson and Puplampu,^28^ suggesting that children’s interactions with digital technology may also affect their cognitive development during the formative years. As such, technology constitutes an ecological subsystem within the child’s environment. This implies that phenomena such as screen use among children must be both understood and addressed from a systemic perspective to capture the numerous factors affecting children’s digital habits. According to the model, it is not a single factor that determines how screen use impacts children and adolescents, but rather the interaction between different levels of their environment. For instance, at the micro level, children’s screen habits can be linked to their parents’ screen behavior,^29^ while at the macro level, restrictions and regulations, such as during COVID-19, can be seen as influencing children’s screen use.^30^ Complementing this perspective, habit loop theory^31^ offers insight into how screen use may become embedded in daily life through recurring patterns of cues, routines, and rewards. These mechanisms help explain how behaviors initiated in specific contexts can be reinforced over time, further emphasizing the interplay between individual behavior and environmental conditions.

Given these interrelated influences, it is essential to incorporate parental involvement in interventions addressing problematic screen use. Parental engagement can play a pivotal role in reducing children’s vulnerability to adverse outcomes across multiple developmental contexts by guiding them toward healthier and more balanced screen use habits through structure, support, and ongoing dialogue. In particular, research has shown that parent–child relationships, including adolescents’ openness and information sharing with parents, are closely linked to the severity of problematic gaming behaviors, underscoring the importance of fostering open communication within the family.^32^

Importantly, research indicates that parents not only express a desire for greater involvement in interventions addressing their children’s problematic gaming but constitutes healthy and age-appropriate screen behaviors.^33,34^ In this regard, strengthening parents’ digital literacy—understood as the ability to critically understand, evaluate, and guide children’s use of digital technologies—appears to be an important complement to efforts focused on regulating and managing screen behaviors^35^ Previous research also shows that parents experience uncertainty regarding both the appropriate amount of screen time and how best to respond to their child’s screen use Previous research also shows that parents experience uncertainty regarding both the appropriate amount of screen time and how best to respond to their child’s screen use.^36^ This highlights an urgent need for evidence-based educational programs for parents, in order to improve their understanding of the risks and benefits associated with screen use and to support more informed decision-making.

The aim of this qualitative study was twofold. Firstly, it sought to explore parents’ perceptions of the impact of their children’s screen use on relationships, school performance, and overall well-being. Additionally, the study endeavored to examine parents’ perceived need of support in managing their children´s problematic gaming and excessive screen use. The questions that this study aimed to answer were as follows:

1) How does children’s screen use impact their relationships, school performance, and well-being, as perceived by their parents?
2) What are parents’ experiences and needs regarding support for managing their child’s screen use?

## Design and method

### Procedure

When planning this study, several possible designs were considered. Given that the subject touches on potentially sensitive areas for the study participants, such as family interactions, data collection through focus groups was excluded in favor of individual and semi-structured interviews.^37^ Members of the research group have longstanding experience in both clinical and academic aspects of problematic gaming and excessive screen use in children. We recognize that this may influence how the collected data is interpreted and analyzed. Therefore, we have taken this into account in the analysis process - see the Data Analysis section for further details.

The interview guide was developed based on the study’s research question to ensure alignment with the study objectives. Interviews were conducted between October 28, 2024, and January 9, 2025, and consisted of 22 questions (4 closed, 18 open-ended) (see Appendix 1 and 2). The primary aim of these questions was to explore parents’ needs and preferences regarding the content of an intervention for problematic gaming and excessive screen use. Each interview was conducted via telephone and lasted an average of 30 minutes (Table 1). The interviews covered various topics, including descriptions of the child’s gaming habits and screen use, as well as their impact on family relationships, education, and leisure activities. Additionally, the interview manual included questions about parents’ recommendations for the content of a possible intervention addressing problematic gaming and excessive screen use. The interviews concluded with an opportunity for participants to provide additional comments and reflections.

**Table 1.** Participants.

Interview person	Parent gender	Child gender	Child age	Interview type	Previously received care	Interview time (minutes)
IP 1	Man	Boy, Boy	14, 18	Phone call	Yes	28
IP 2	Woman	Girl, Girl	16, 17	Phone call	No	35
IP 3	Man	Girl, Girl	16, 17	Phone call	No	33
IP 4	Woman	Girl, Boy, Boy	13, 15, 17	Phone call	No	42
IP 5	Woman	Boy, Boy	12, 15	Phone call	No	39
IP 6	Man	Boy, Boy	12, 15	Phone call	No	16
IP 7	Woman	Boy, Girl, Boy	12, 14, 16	Phone call	No	32
IP 8	Man	Boy, Girl, Boy	12, 14, 16	Phone call	No	35
IP 9	Woman	Boy, Boy	13, 16	Phone call	No	25
IP 10	Man	Boy, Boy	13, 16	Phone call	No	17
IP 11	Woman	Boy, Boy	16, 17	Phone call	No	27

Prior to participation, informed written consent was obtained from all participants in accordance with ethical research guidelines. The study was approved by the Swedish ethical review authority (EPN, DNR 2023-05533-01; 2023-09-19 024-05739-02; 2024-10-05).

### Participants

Participants for this study were recruited from five schools (age group 12-18 år) in Lund, a medium-sized Swedish university town. Approximately 1,200 households were given the information on recruiting. Initial contact was established through school principals or school healthcare teams. The last author distributed a written invitation along with comprehensive information about the study, followed by a subsequent telephone call to ensure clarity and encourage participation. The schools then disseminated the written study information to all guardians, inviting them to contact the research team if they were interested in participating. In this study all guardians were also parents hence here after named parents. The inclusion criteria required that participants be caregivers of children and adolescents aged 12 < 18 years who reported experiencing familial difficulties related to the child’s screen use. Additionally, participants were excluded if they had ongoing engagement with outpatient child and adolescent psychiatric clinic. A total of 17 parents expressed an interest in participating in the study, of whom 14 met the inclusion criteria. Ultimately, 11 parents, comprising six women and five men, were interviewed. In three cases, both parents of a child participated, with mothers and fathers interviewed separately. In these cases the parents were separated and lived in different households.

### Data analysis

After nine interviews, participants’ responses began to show strong similarities, and no substantially new insights emerged. To strengthen the robustness of the findings, two additional interviews were conducted. Each interview was digitally recorded using a portable dictaphone (OLYMPUS VN-541PC). . Transcriptions were conducted verbatim through careful listening to the recordings and manually documented in a Word file. The primary purpose of transcription was to facilitate data analysis and enable comparisons between participants’ responses to identify main themes and subthemes.^38^ Upon completion, all transcriptions underwent a thorough review and quality check to ensure accuracy. Data analysis was conducted following the thematic analysis framework outlined by Clarke & Braun.^39^ This method was chosen due to the study’s inductive approach, as it can help identify patterns in parent’s responses.

The three authors who analyzed the data have a substantial breadth of prior experience in the researched problem area, which we believe has contributed to a comprehensive and nuanced analysis process. The process involved familiarization with the material to gain an understanding of its contents. Relevant excerpts from the original transcriptions were systematically extracted and organized into sentence units, representing concise descriptions of participants’ responses. These sentence units were then interpreted and coded, creating initial themes. The initial themes were reviewed, and the material was analyzed. The categorized sentence units were compared across participants to identify recurring patterns and similarities, which were subsequently grouped into subthemes. These subthemes were further synthesized into overarching main themes, reflecting the core perspectives expressed by the participants. The main themes and subthemes were defined and titled. All interviews were conducted in Swedish, and the findings were later translated into English for presentation in the results section. The translations were carried out by the researchers and jointly agreed upon to ensure that the wording remained as close as possible to the original Swedish text.

## Results

The thematic analysis yielded three main themes and nine subthemes (see Table 2). Theme 1 - Consequences of children’s screen use, generated the four subthemes’ relationships, education, health, and development, with further subcategories. Theme 2 - A problem for the whole society, resulted in the two subthemes what society should focus more on and what society should regulate. Theme 3 - Support on family and individual level, generated the three subthemes an external expert, support for youth and support for parents.

**Table 2.** An overview of main themes and subthemes.

Theme	Consequences of children’s screen use	A problem for the whole society	Need of support on family and individual level
Subtheme	Relationships^2^	What society should focus more on^3,4^	An external expert^2,3^
	Consequences for family dynamics		
	Relational consequences parent-child	What society should regulate^4^	Support for youth^3^
	Education^1^		
	Positive consequences for the child’s education	​	Support for parent^3^
	Negative consequences for the child’s education		​
	Health^1^		
	Consequences for physical and mental health		
	Development		
	Concerns about the long-term consequences		

The table also shows how each theme relates to Bronfenbrenner’s bioecological model: 1) Microsystem, 2) Mesosystem, 3) Exosystem, 4) Macrosystem.

### Consequences of children’s screen use

The first main theme illustrates the consequences that screen usage in children has on their relationships with their parents, how it impacts their education, health and development. Most of the parents reported encountering various challenges in managing situations arising from screen use and expressed a need for intervention.

#### Consequences for family dynamics

This subtheme describes how family dynamics are influenced by the child’s screen use. All parents in this study agreed that excessive screen use had an impact on family dynamics. Several parents reported on their children becoming more passive, communicating less, and spending less time with their family.

“It affects the interaction because we have let go of a lot of control […], mainly to avoid conflicts. However, this results in less interaction and communication within the family […], when they spend a lot of time on screens or have headphones in and prefer to eat alone, for example.” -IP6

The essence of the statement above is that to avoid conflict the parent has eased screen time restrictions. While this has led to reduced interaction and communication with the family, it is viewed as a necessary compromise to maintain harmony.

“You’d rather sit in bed and mindlessly scroll than spend time with family and friends” -IP4

The parent explains with an emotional sense that the child prefers to spend time with digital devices than to spend time with the family and friends. Furthermore, it had an impact on the relationship between parents. One mother stated:

“It affects the relationship between my husband and me as well, because there’s a lot of arguing at home. And I feel that I’ve been more involved than he has.” -IP6

The underlying meaning by the quote above may be that the children’s excessive screen time affects the family dynamics and that the increased use of devices generates more conflicts at home, which in turns impacts the relationship with her husband. The mother also reflects on being more involved in addressing and handling these situations than her husband and seeks support.

Several parents reported being willing to control what their children are doing on the internet using applications that allow parents to monitor and control their children’s online activity across various digital devices. Parents can review search history, set screen time limits, block, or approve app downloads. Additionally, to ensure that only age-appropriate content is accessed, parents can apply filters to help them create a safer online experience for their children. One parent stated:

“We’ve tried to limit her screen time using the Family Link app, both in terms of when she can use it and how much. […] We ended up having to face the conflict, and it doesn’t settle until she gets her way!”-IP7

The quote describes that when parents try to set boundaries and limit their children’s screen time it provokes huge conflicts. These conflicts affect the family dynamics and leave the parents powerless and without effective strategies to handle the situation. The parent continues,

“If we turn off the phone, she’ll just take the computer instead. If we turn off the computer, it’s total chaos, and then there are such big conflicts that it affects the whole family.”-IP7

The above quote clarifies that the parent is frustrated and that various attempts to set boundaries and limit screen time result in a vicious cycle of conflicts that impact the entire family dynamic. Meanwhile, some parents try to find a balance between digital development and their own expectations. One parent stated:

“We have set limitations on the phones. And then there’s a lot of nagging. So, the consequences are bad moods and poor relationship. That’s why it’s difficult. I find it hard to manage because, on one hand, this is how the future is. […] But at the same time, you have a different idea of how you would like things to be, which you try to maintain.”-IP5

The parent describes something very important, his statement highlights that he tries to maintain rules for healthy screen time but struggles with the consequences that follow, such as bad moods and negative impacts on relationships. In this context, parents express a desire for an intervention that could help manage these challenges more effectively.

#### Relational consequences for the parent-child relationship

The second subtheme describes the various ways the relationship between parents and children are affected due to excessive screen use. Some parents describe struggles in connecting with their children, arguments, and constant conflicts, while other parents explain that they need to be vigilant and monitor their children’s screen usage. These things result in a negative atmosphere and emotional outbursts. Several parents struggle to find effective strategies and solutions to the problem. One parent stated:

“I become controlling and perhaps interfere in a way that I don’t think is appropriate for a child of that age”-IP11

The essence of the quote above is a self-reflection of a parent that realizes the behavior of being controlling and in a way overstepping the child’s boundaries can have a negative impact on their relationship. The parent is questioning if her actions can be considered appropriate and healthy in relation to the child’s age while also trying to maintain control and set boundaries. Furthermore, with the increasing use of digital technology, parents are faced with growing concerns and challenges in managing their children’s online activity to ensure their safety and emotional wellbeing. Consequently, it impacts the relationship between the parent and the child.

“It feels like I’m always chasing after them, constantly on their case. What are you doing now? It’s like I’m always having to keep an eye on them- are you really doing your homework?”-IP6

The parent express frustration and a constant need to control the children’s online activity. It highlights the balance between trusting one’s children and the urge to monitor their behavior which, over time, can negatively impact the relationship between the parent and the child. Some parents removed all restrictions regarding excessive screen time to avoid the relationship between the parent and child from deteriorating. One parent stated:

“It became such a hassle just around the use of the phone. Every conversation turned so negative in some way that we felt it wasn’t working anymore, so we let it go”-IP3

The parent describes how excessive screen time restrictions affected their relationship negatively and lead to constant arguments and conflicts. The negative impact outweighed the benefits, and the parents decided to remove all restrictions. Furthermore, parents report that growing up without digital tools make it hard to understand the modern worldview. The parent continues:

“Yes, they live in some sort of yes completely in their own world, entirely in their TikTok world” -IP3

The above sentence reflects that the parents feel the child lives in a different world than the parent that the parent has difficulties in understanding. The parent also indicates that it is hard reaching your children and starting conversations because their attention is being occupied by social media platforms.

#### Positive consequences for the child’s education

This subtheme illustrates the positive outcome from interacting with different digital tools in education. All the parents in the study experienced something positive from incorporating digital tools in education.

“They are incredibly quick to Google something whenever they don’t know how to do it”-IP4

The underlying meaning of above quote is that the development of digital devices in the education system have made it easy to solve problems in the daily lives. Several parents report that their children have become more creative, learned facts, and became more knowledgeable after interaction with digital devices. There is a wish to maintain the positive benefits from learning without leading to problematic gaming and excessive screen use.

Nevertheless, there are also students that use different social media platforms to discuss homework. If you are not available, you risk missing out on important information. The parent continues:

“Yes, I think the educational value of Settera is fantastic […] he can set out all the states, all the capitals, and all the capitals across the entire states of America. He can set out all the countries and capitals from practically all over the world, and that’s because they’ve designed such activities.”-IP4

The parent values the educational impact that the platform Settera have on her child. Settera is a platform for learning geographic facts through various of exercises and quizzes. Providing increased knowledge and education.^40^

“An example would be vocabulary words. Instead of having a book with the words, they are listed on the school’s website, where you can go in and read them. Additionally, there are useful features like a program where you can practice writing the words and check if they’re spelled correctly, which I find to be quite educational and well-structured. “-IP6

The quote describes how the digital system works to support vocabulary practice for educational purposes. Rather than relying on traditional books, the vocabulary words are provided on the school’s platform, enabling students to receive instant feedback on their spelling. It empathizes efficacy and enhance learning.

“When it comes to audiobooks, it’s almost always a positive experience. It’s like reading books, but you can do it while multitasking” -IP1

The statement highlights the benefit of listening to a book while multitasking. The parent reflects on how modern reading methods, such as audiobooks, don’t demand the same level of time and focus as traditional reading.

“One thing that has greatly improved since they started using their phones is their English”-IP3

The essence of the above quote focus on how the use of mobile phones have a positive impact on language skills. Reading in English, engaging in different content provided in English enhances the ability to become bilingual.

“He is the most knowledgeable of my children […] he is always updated on what’s happening in the world” -IP11

Notably, increased screen use can provide positive benefits for education. It can enhance knowledge about the world and language, and there are numerous websites and applications that can aid in improving learning. There is a wish to maintain educational value of digital devices in education system meanwhile decreasing overuse and digital distractions.

#### Negative consequences for the child’s education

This subtheme explains the negative consequences of digital devices in education and emphasizes a need for an intervention. Most of the parents in this study indicated that their children managed their education well, while some children were reported having trouble waking up during the morning because of late screen activity, resulting in coming late to school. The digital devices are integrated in the school system and all education is based on having a computer. The student’s assignments are being presented on the digital platform.

“Schoolwork is based on doing everything on the computer, and there are no textbooks anymore”-IP5

The parent reflects on the integration of digital devices in the education system. The parent continues:

“And then it’s easy since there’s no barrier. You look at the school assignment you’re supposed to be doing and then you have 13 other tabs open with different things, so you switch to those. It ends up stealing your focus from the task at hand”-IP5

Several parents shared the same approach on the integrated digital system in education. The parent reflects on how easy it is for the child to shift focus from schoolwork to other websites that may feel more engaging and underlines a need for support. It requires discipling and motivation to stay on track.

“He uses his school laptop and lies in bed watching YouTube. When you walk in, it’s obvious that he’s not working on his school tasks.” -IP11

Notably, it’s challenging to fully concentrate on their schoolwork while having a Chromebook that makes it easy to shift focus to other platforms like YouTube a popular platform for sharing videos.

“But I would say that it’s problematic having schoolwork on a computer. You can’t just say, “Sit down and do your homework, close the door.” As a parent, you constantly must monitor to make sure they don’t get distracted by something else.” -IP6

The statement explains that it requires the parent to be vigilant and constantly monitor the children ensuring that they do their homework. The challenge for the parent is to prevent the multiple distractions on the digital environment. The parent continues:

“Just yesterday, I saw that he was supposed to be doing his English homework, but he was just playing games. That’s also a frustrating area—the vocabulary is online. […] So, you can’t do your homework without using digital tools. I find that annoying.” -IP6

The involvement of a parent in the children’s education is significant and requires more attention and dedication. The parent empathizes a problematic area that evokes frustration and disappointment.

Furthermore, some schools prohibit mobile phones during lesson time while others allow them for educational purposes or during breaks. Some parents argued for an impact on reducing the attention span, impacting focus and steal their time from school assignments. Among the parents in the study there were a growing concern that children expect thing to happen quickly, demanding instant gratification and making it harder to focus on tasks that require sustained effort. This reinforces the desire for an intervention to help address these challenges.

#### Consequences for physical and mental health

The integration of digital technology evokes concerns regarding physical and mental health among the parents in the study. Most of the parents agreed on one thing, they believe that the children would have been more active in recreational activities if they hadn’t been so active on digital devices. The parents seek to find a balance between leisure activities and overuse of digital devices.

“I think the balance became worse when the activity disappeared, so I feel it’s more of a change, quitting in the association, that contributes to the balance getting worse for us” -IP11

The parent is reflecting on the impact of leaving recreational activities and replacing it with a more passive activity that have an impact on health and lifestyle. The difficulties some of the parent’s face is trying to find activities that are as motivating as digital devices.

“Yes, but the problem is that they don’t develop social relationships in the same way. They become more sedentary, with poor posture from staring at their phones. […]. It also gives them a distorted view of realty- seeing YouTubers and influences showcase the best part of their lives, and then believing that’s the way things should be” -IP4

It captures the parent’s perception of the various impacts screen time on social, mental, and physical health. The parent reflects on how it affects relationships, leads to a lack of social skills, and creates a distorted sense of reality. It contributes to an unrealistic view of the world. The parent further discusses the significant physical consequences, such as poor posture, as well as cognitive effects, including reduced attention span and difficulty focusing on slower-paced tasks. Meanwhile other parents are more concerned that their children sleep quality is being affected by their screen time.

“She feels bored when she’s at home, so she turns to her screen, leading to excessive screen time. As a result, she has trouble falling asleep, which makes it hard for her to wake up in the morning, leaving her too tired to go to school. It becomes a vicious cycle” -IP7

The statement reflects the parent’s frustration with their child’s screen time and the resulting fatigue which impacts their ability to focus in a school setting. Breaking this cycle is challenging, as the parent lack effective strategies. While most parents acknowledged some positive aspects of socializing through screens, they also felt that not socialize with others online was a negative aspect. Some parents reflected on personality as being an important factor for influence on digital devices.

“It’s not really about the age, but personality. How easily one is influenced by different things”-IP9

The underlying meaning of this quote is that there may be a vulnerability in different people. In other words, personality and the degree of influence are more important than age when it comes to assessing how a young person will react and handle social environments and highlights that interventions should be individualized.

“They manage school well and spend a lot of time with their friends, which is maybe why we’ve decided to ease up on it, because they handle extracurricular activities and things like that.” -IP2

The sentence above were a common theme among parents in this study, as they felt that they could reduce or completely remove restrictions on digital devices if their children managed their studies and extracurricular activities. Although it still bothered parents that their children spent excessive time on different screens and got absorbed by it, it somehow highlights that there is a certain acceptance of screen use, given that the children manage their commitments.

Various interpretations were described to make the situation reasonable. Most parents in the study described their children’s screen time as an addiction while other parents associated it to their child’s mental health and general functioning.

“Well, that’s exactly it- you almost become addicted to the constant stimuli from your phone, so when you’re not getting that simulation all the time, you experience a kind of boredom that they can’t really cope with”-IP1

The parent reflects that the children become addicted to the constant stimulation of their mobile phones. The underlying meaning of above quote is that the digital world, through social media platforms, games, and other applications, keeps the user constantly engaged and filled with stimuli. When this stimulation is absent, it creates feelings of frustration and discomfort.

“And how do you help someone who is dependent on an addiction to something that is available everywhere, all the time?”-IP6

This statement describes the child’s screen use as an addition which evokes frustration and helplessness, underlining the desire for an intervention to address the serious concerns this bring to the family.

#### Concerns about the long-term consequences

This subtheme demonstrates the parents’ concerns about the long-term effects of screen use on attention, focus, social skills, and relationships, with a specific concern about their children’s ability to handle future responsibilities. Significantly, there is a need for an intervention to address the impact of screen usage on mental and physical health,

“If there’s one thing, we know for sure, it’s that physical activity is important. Whether the media is the cause of sitting, or if it’s something else, I’m not certain, but either way, being so sedentary is not good” -IP11

The parent reflects that excessive sitting and not being active is believed to have harmful effect in the long term. Meanwhile, other parents are concern about how their children will cope in the future when they no longer have their parents to provide support.

“How does it work in school then? Because there you have at least a 40-minute lesson. […] But I’m also thinking ahead. How will this work? When they’re living on their own and I’m not there to push them to handle the work”. -IP4

This quote illustrates the parent’s concern about the child’s future ability to manage tasks that require time management and focus. This could be challenging if the child is accustomed to an environment that offers instant gratification and fosters a short attention span due to excessive screen use.

### A problem for the society

The second main theme aims to explore the interventions and support parents in the study are seeking from society, as well as the regulations society should implement to prevent harmful screen use. A significant proportion of parents report that they have not sought support and are unaware of where to turn for guidance.

#### What should society focus more on

The overall view from the investigation is that the parents wish society would engage more in the issue of excessive screen time. Most of the parents in the study agreed that it was a societal issue and wish for the society to contribute to addressing this issue.

“It there were extracurricular activities for children struggling with gaming addiction or excessive screen use […] it would be worth every penny to help these young people get out, get back on their feet, and eventually be able to live a meaningful life” -IP2

The parent emphasizes the importance of providing children who struggle with overuse of screens the opportunity to engage in extracurricular activities. They believe that investing time and resources in such initiatives is worthwhile for improving the children’s overall well-being.

“But also, in some way, the importance of having a non-digital hobby. You might have one when you're younger, but eventually, many stop […] The key is having something non-digital that can help create balance.” -IP11

Notably, the parent highlights the importance to have a hobby that fosters a non-digital environment that contributes to a more balanced lifestyle.

“It doesn’t help that my children put away their phones! It is a societal issue!”-IP2

The parent reflects on the issue with the approach that it isn’t enough for just her children to put away their phone, but that it’s a bigger problem, a societal problem. However, parents were united in the opinion that leisure time should be more balanced, and the society should offer teenagers recreational activities to limit the use of digital devices. They were convinced that the society should take responsibility and is better supplied to handle the situation.

#### What should society regulate

This subtheme explores the regulatory measures society should consider to alleviate the negative effect of screen use. Parental recommendations included recommendations for public health authorities, the establishment of age restrictions on social media platforms regulated by the government and limiting access to digital devices within the education system, all aimed to reduce unrestricted digital use.

“Recently, the Public Health Agency issued recommendations regarding screen time. I think, in a way, it’s quite helpful because it allows you to say, ‘We don’t have any other rules in our family beyond what the Public Health Agency recommends.’”-IP5

The statement describes support from an authoritative source as the public health agency which provides parents support in explaining and justifying decisions regarding screen time use. It works as a rational for parents and a possibility to reduce conflict regarding screen time limitations and fosters healthy relationship within the family. Furthermore, there is also an aspiration to limit screen use in education.

“Yes, initially, I believe the entire society would benefit from support. For example, we should remove screen from schools. There shouldn’t be a need for computers at all”-IP2

The parent requires limited access to digital tools in the educational system, an aspiration to shift the societal norms. The aim to reduce digital access allows for a more traditional way of learning and potentially enhance the benefit from interacting with each other.

### Support on family and individual level

The third main theme investigates the support parents seek from healthcare and community services to promote and sustain healthy screen habits for both the family and the child. One common aspect shared by most parents in the study is that they have all tried to implement various strategies and rules, which has yield different outcomes.

#### An external expert

This subtheme examines parents’ request for external professional support, potentially from healthcare providers or local community services, to offer children and adolescence the opportunity to discuss excessive screen use within the context of established, clear guidelines and boundaries.

“I believe it would have been valuable for the young people to engage in the discussion themselves. It’s challenging for the adult world to regulate this. There should also be support for the parent but it’s the discussions that leave an impact.”-IP8

The statement emphasizes the importance of providing children and young adults with an environment that facilitates the development and practice of various strategies, enabling them to maintain a healthy relationship and balance between digital use and daily life. Although, when seeking help from the municipality it came with mixed emotions.

“We eventually sought help from the municipality. It was them who said that we couldn’t set any demands, […]. That’s when it got even worse, and he was given a free pass. That’s when things went from bad to really bad.”-IP1

The essence of above quote explained the experience the parent had when seeking help from the municipality and the parent felt that the situation got worse rather than improving. The parent interpretated the recommendations as not providing enough of structure or guidance for the child or the parents. The child was given more freedom without responsibility, resulting in a negative development. The parent continues,

“They wanted to get to know everyone in the family on an individual level. Then try to move on to some sort of action. But there was no real connection in between […]. So somewhere there should be something like, ‘This tends to work,’ or maybe a toolbox with tools.”-IP1

This statement describes an aspiration to have several proven methods or a defined action plan, that parents can use when dealing with children with excessive screen use.

#### Support for youth

This subtheme describes what parents in the study believed their children needed in term of support for excessive screen use. In summary, parents in the study express a desire for psychological support and the chance for open, trust-building conversations. But above all, the opportunity to extracurricular activities and to create healthy social relationships.

“I think about those who have problematic gaming and excessive screen use, they probably lack friends and don’t know how to make connections. […] We put a lot of thought into how we could help our oldest daughter find friends. Eventually, we considered options like going to church or other places. It’s not easy for teenagers. They have school, but if they don’t connect with their peers, what are they supposed to do?” -IP2

The parent reflects on how to help and support her teenager and how difficult it is for young people to find their place in different contexts. By enhancing opportunities for children to connect with friends, there is hope that this will reduce excessive screen use.

“What happens when you use screens so much? And what do you miss out on? Because if you’re always on your phone, you’re missing out on meeting friends, going to school and a lot of things that also is fun” -IP7

The parent reflects on the discussion between herself and her child, expressing a desire for an intervention addressing overuse of screens. This intervention could involve discussion groups with individuals facing similar issues, facilitated by an external professional, potentially within a healthcare setting. Such a professional could help structure the discussions, provide guidance, and offer a framework for the intervention. Additionally, the discussion would focus on the impact of digital devices on individuals and the consequences of screen use.

#### Support for parents

In this subtheme, parents describe the support they need in their parenting to manage excessive screen use. We encountered a variety of parents, each with their own approach to addressing the issue. As a result, the parents aspire to a support system that is tailored to enhance knowledge and facilitate meaningful discussions.

“I wish that, as an adult someone had told me […] I find it hard to determine. When is it an addiction? Is it an addiction or is he just a teenager? When is it a problem?”-IP4

The sentence above emphasizes a parents worry and frustration to not be able to determine where the line is between normal teenage behavior and addiction. The parent is requesting support to better understand this.

“I would have appreciated an easily accessible website with a clear, pedagogical brochure – something that could offer guidance on what to consider, what’s important, and how to approach things as a parent. A form of support to help maintain routines and practice different strategies.” -IP11

The parent reflects that information should be easily accessible and easy to understand, providing parents with concrete tips and guidance for making decisions in everyday situations. This includes support in establishing routines and practicing strategies to manage excessive screen use.

“We participated in a parent education program organized by the municipality, called ABC Teenagers. During the program, there was considerable discussion with other families, where everyone shared some of their challenges and approaches to resolving them. This was inspiring. […] It also fostered a sense that we are not alone […]. I believe that something similar—organized discussions involving teenagers—would also be beneficial.” -IP7

The sentence emphasizes positive experiences with a municipal parenting program and highlights the value of group discussions. Such discussions can provide new perspectives, enable the exchange of experiences, and reduce feelings of isolation. The parent also suggests an improvement, namely, to include the teenagers in similar forums.

“But also support for the parents. I don’t believe that simply watching a film is effective; it is the discussions that truly make an impact.” -IP8

Notably, the parent reflects on the need for more active support for parents and questions passive measures, such as merely showing a film. What is perceived as most meaningful and effective, however, are discussions and conversations where participants have the opportunity to share experiences with one another.

## Discussion

The aim of this qualitative study was to explore parents’ perceptions of the impact of their children’s screen use, and to examine their perceived need for support in managing problematic gaming and excessive screen time. These findings are discussed below in relation to previous research in the field and Bronfenbrenner’s ecological systems theory of individual psychological development, to provide a deeper understanding of the study’s results.^27,41^

Children and adolescents are growing up in a world where technology dominates many aspects of their daily lives—a world that parents may find difficult to fully understand.^36^ Parents in this study highlighted both the benefits and drawbacks of children’s screen use, noting, for example, that digital devices can support learning, social interaction, creativity, and access to information, while also noting the problematic aspect of children in Sweden having access to digital devices through compulsory schooling—devices that are often used for purposes beyond education. Access to school-provided computers is an example of how decisions made at the macrosystem level affect the individual, while the individual has limited ability to influence these conditions. While the integration of digital devices into the school system facilitates access to learning platforms, digital tools can also strengthen students’ engagement and offer alternative ways of demonstrating knowledge. Parents acknowledged that technology can be particularly beneficial for children who struggle with traditional learning formats or who use digital platforms to maintain friendships and build social competence. However, the increased reliance on digital resources also heightens the need for skills in source criticism, as children often tend to trust information found online.^42^ Parents in our study acknowledged the educational benefits of digital tools such as audiobooks and vocabulary apps that provide immediate feedback. However, some expressed concern about how digital devices are used in schools, arguing that overuse may reduce children’s attention spans and shift focus away from schoolwork. This was seen to hinder engagement with slower-paced learning activities, consistent with findings in previous studies.^43^

While the macrosystem sets important conditions, many of the day-to-day challenges take place within the family context. This study indicates that families frequently struggle with conflicts related to excessive screen use. Parents reported reduced communication within the family and increased sedentary behavior among children. The findings also point to social and cognitive consequences of screen overuse. Several parents used the term “addiction” to describe their children’s screen behavior, though many were unsure whether it reflected a genuine problem or a normative part of growing up. This uncertainty sometimes prevented them from seeking professional help. Although such descriptions do not necessarily indicate addiction in the clinical sense,^44,45^ the terminology reflects the seriousness with which some parents perceive the issue. The frequent use of the term “addiction” may also reflect how parental understandings are shaped by broader public discourse, where excessive screen use is often framed in biomedical or alarmist terms. Media narratives and societal debates frequently employ addiction language when describing children’s gaming and social media use, which may influence how parents interpret normative developmental behaviors. Adolescence is characterized by heightened engagement in peer-oriented and reward-driven activities and distinguishing between developmentally typical intensity and clinically significant impairment can be challenging. The use of addiction terminology may therefore signal parental uncertainty, concern, or a search for conceptual tools to make sense of behavioral changes, rather than a clinical judgment per se. This highlights the importance of providing parents with nuanced psychoeducation that differentiates between high engagement and problematic use. A more refined public and clinical discourse may help reduce polarization between normalization and pathologization of young people’s digital lives.

Parents in this study expressed a desire for interventions that help restore balance between digital use and other leisure activities. On a broader level, they emphasized the importance of greater societal engagement in supporting children who struggle with excessive screen use, including improved access to extracurricular activities in non-digital settings. At the same time, parents’ reflections tended to focus primarily on structural and societal solutions, while less attention was given to their own role in initiating or sustaining children’s participation in such activities. The concurrent call for greater societal support to engage children in non-digital leisure may therefore appear paradoxical and warrants further investigation to understand the underlying mechanisms and perceived barriers Consistent with previous research,^46^ many parents also emphasized the need for clearer societal regulations, such as age restrictions on social media platforms and limits on screen use in educational settings. At the same time, parents also recognized the benefits of digital interaction. Some expressed satisfaction that their children were socially engaged online, reflecting the potential advantages that have also been highlighted in previous studies.^47^ This suggests that parents hold a nuanced view of social media use, acknowledging both its potential benefits and its drawbacks.

Parents in this study expressed a need for guidance on when to seek help and how to talk to their children about the physical and mental health consequences of excessive screen use. Some found public health recommendations helpful in navigating these challenges.^48^ As in previous research,^33^ parents expressed a preference for receiving support from professionals with specific expertise. Interestingly, our findings do not suggest that parents themselves emphasize the need for joint interventions involving both parent and child—such as family therapy—to address strained family dynamics. This is noteworthy given that many parents describe deteriorating relationships as a consequence of children’s screen use. One possible interpretation is that family-based interventions are not perceived as relevant or accessible, despite an awareness of negative impacts on the family climate.

The findings of this study suggest the need for differentiated intervention strategies operating at multiple levels. At the parental level, support may include accessible psychoeducation that helps parents distinguish between normative and problematic screen use, practical guidance on boundary-setting, and structured opportunities for peer discussion with other parents facing similar challenges. At the child and adolescent level, interventions may focus on motivational conversations, strengthening self-regulation skills, promoting alternative leisure activities, and facilitating balanced digital habits without pathologizing normative engagement. In families where screen-related conflicts have strained relationships, family-based approaches that address communication patterns and shared rule-setting may also be beneficial. At a structural level, parents’ narratives point to the importance of clearer public health guidance, school policies regarding digital device use, and increased availability of non-digital extracurricular activities. Distinguishing between these levels of response may support the development of interventions that are both context-sensitive and aligned with the ecological systems in which children’s screen use is embedded. While interventions for social media addiction and internet gaming disorder have shown promise in reducing symptoms and improving mental health,^44,49^ there is a continued need to develop approaches that involve the family. Parents play a central role in the child’s microsystem and are key actors in shaping the conditions for sustainable behavioral change.^50^ From the perspective of habit loop theory,^31^ these findings highlight how children’s and adolescents’ screen use is sustained through recurring interactions between cues, routines, and rewards embedded in everyday family life. Interventions that engage parents and families may therefore be particularly important in disrupting maladaptive habit loops and supporting the development of more adaptive and sustainable digital habits by jointly reshaping contextual cues, everyday routines, and the meaning attached to digital rewards. Taken together, these findings underscore the need for multi-level interventions that combine structural measures with accessible, family-oriented support strategies aimed at promoting healthy digital habits in children and adolescents. Such interventions should also strengthen parents’ digital literacy,^35^ enabling them to better support their children in developing balanced, informed, and sustainable screen use practices that promote overall screen health.

Sweden is characterized by a highly digitalized school system, widespread access to personal digital devices, and a strong welfare infrastructure that shapes both parental expectations and available support structures. For example, the integration of laptops in compulsory schooling and the presence of publicly funded leisure activities may influence how parents interpret both the risks and the societal responsibility for managing children’s screen use. In contexts with lower levels of digital integration in education, different regulatory frameworks, or less accessible welfare and extracurricular systems, parental perceptions and support needs may differ substantially. At the same time, the tensions described by parents—such as conflicts around boundaries, uncertainty about what constitutes problematic use, and calls for clearer societal regulation—are likely to resonate beyond the Swedish setting.

## Conclusion

Our conclusion is that, according to parents, screen use among children is a complex phenomenon that calls for broad and systemic responses. While consequences for the child are primarily observed at the microsystem level—affecting relationships, health, and development—parents express expectations for change at higher levels, particularly the macrosystem. This includes public regulation of digital media and greater access to alternative leisure activities. This observation may offer a valuable perspective, as it raises questions about the approach of interpreting behaviors like excessive gaming or social media use through a medical, and more individual, lens—such as through the formalization of excessive gaming as a medical diagnosis. Rather than viewing screen-related challenges solely as a family responsibility, parents often see them as embedded in broader structural and societal contexts. This insight is crucial for actors working to support families navigating children’s problematic screen use.

## Limitations

The findings reported in this study should be considered in light of several limitations. First, the data reflect parents’ self-reported experiences, which may be influenced by subjective bias and may not fully capture children’s actual screen use or its impacts. The qualitative design, using semi-structured interviews, enabled in-depth exploration of parental perspectives but also introduced variability in the topics discussed, potentially affecting consistency across interviews. Second, the study focuses exclusively on parents’ perspectives on excessive screen use, without incorporating the views of children or adolescents. This limits the scope of the findings and may result in an incomplete understanding of children’s experiences and behaviors. Third, the study is subject to potential selection bias. Parents with strong opinions or concerns about their children’s screen use may have been more likely to participate, while those with fewer concerns may be underrepresented. In addition, although recruitment through five schools reached approximately 1,200 households, only 11 eligible participants were included. This low response rate raises concerns about the effectiveness of the recruitment strategy and the representativeness of the sample. Fourth, the sample lacked sufficient diversity, and detailed demographic information (e.g., socioeconomic status, parental education, migration background, and geographical location) was not collected. This limits the ability to assess how structural and socioeconomic factors may have shaped both children’s screen use and parental perceptions, and it restricts the transferability of the findings. Finally, the study is situated within a Swedish context characterized by a highly digitalized education system, widespread access to personal digital devices, and publicly funded support structures. These contextual conditions may influence parental expectations and experiences and limit the applicability of the findings to settings with different levels of digitalization, regulatory frameworks, and welfare support.

Recent studies have explored the perspectives of children and adolescents regarding problematic gaming.^51^ Future research should incorporate both parents’ and children’s perspectives, recruit more diverse samples, include comprehensive demographic data, and consider longitudinal designs to better understand how children’s screen use and its impacts evolve over time.

## Supplemental material

Supplemental material - Parental perspectives on children’s screen use: Exploring impact, challenges, and support needs - A qualitative study Supplemental material for Parental perspectives on children’s screen use: Exploring impact, challenges, and support needs - A qualitative study by Marie Werner, Felicia Delfechresh, Aleksandra Barstowe, Sabina Kapetanovic, and Emma Claesdotter-Knutsson in Journal of Public Health Research.

Supplemental material - Parental perspectives on children’s screen use: Exploring impact, challenges, and support needs - A qualitative study Supplemental material for Parental perspectives on children’s screen use: Exploring impact, challenges, and support needs - A qualitative study by Marie Werner, Felicia Delfechresh, Aleksandra Barstowe, Sabina Kapetanovic, and Emma Claesdotter-Knutsson in Journal of Public Health Research.

## Data Availability

The data that support the findings of this study are available from the corresponding author upon reasonable request.*
