# Supplementary material for: Parental perspectives on children’s screen use: Exploring impact, challenges, and support needs - A qualitative study
Source: J Public Health Res. 2026 Jun 17;15(2):22799036261462200. doi: 10.1177/22799036261462200 (PMC13291565; doi:10.1177/22799036261462200)
Supplement: Supplemental material - Parental perspectives on children’s screen use: Exploring impact, challenges, and support needs - A qualitative study [file sj-pdf-1-phj-10.1177_22799036261462200.pdf]

Interview Guide (English Translation)

What are the age and gender of your children?

Can you tell me a little about your child’s gaming/screen use?

- When does he/she use screens?
- How much?
- What does he/she do when playing or using screens?

How do you perceive your child’s gaming/screen use?

- Anything positive about it?
- Anything negative?

What do you consider to be the most problematic aspects of your child’s gaming/screen use?

In what way is it problematic?

- How does the issue affect your interaction with your child?
- How does it affect other relationships, school, leisure activities, etc.?
- How is your child affected by the problem?

When did the problems begin?

How do you usually try to handle these problems? What are the outcomes?

Have you received any kind of support for this issue?

- If yes, what type of support?
- If no, how do you go about seeking support?

If your family had the opportunity to receive support for problematic gaming/screen use, what would that support look like?

- What content would you want it to include?

Do you have any additional comments or reflections?
