# Supplementary material for: Parental perspectives on children’s screen use: Exploring impact, challenges, and support needs - A qualitative study
Source: J Public Health Res. 2026 Jun 17;15(2):22799036261462200. doi: 10.1177/22799036261462200 (PMC13291565; doi:10.1177/22799036261462200)
Supplement: Supplemental material - Parental perspectives on children’s screen use: Exploring impact, challenges, and support needs - A qualitative study [file sj-pdf-2-phj-10.1177_22799036261462200.pdf]

Intervjuguide

Vilken ålder och kön har era barn?

Kan du berätta lite om ditt barns dataspel/skärmanvändning?

- När använder han/hon den?
- Hur mycket?
- Vad gör han/hon när hen spelar/använder skärm?

Hur uppfattar du ditt barns dataspel/skärmanvändning?

- Något positivt?
- Något negativt?

Vad uppfattar ni som mest problematiskt med barnets dataspel/skärmanvändande?

På vilket sätt är det problematiskt?

- Hur påverkar problemet samspelet mellan er och ert barn?
- Hur påverkar problemet andra relationer, skola, fritid etc?
- Hur blir barnet påverkat av problemet?

När debuterade problemen?

Hur brukar ni göra för att hantera problemen? Vad får det för resultat?

Har ni fått någon form av stöd för problemet?

- Om ja, vilken typ av stöd?
- Om nej, hur gör ni för att få stöd?

Om ni som familj skulle ha möjlighet att få stöd för problematiskt dataspel/skärmanvändande hur skulle det stödet se ut?

- Vilket innehåll skulle ni önska att stödet hade?

Några fler synpunkter och reflektioner?
